# Echinacea reduces antibiotic usage in children through respiratory tract infection prevention: a randomized, blinded, controlled clinical trial

**DOI:** 10.1186/s40001-021-00499-6

**Published:** 2021-04-08

**Authors:** Mercedes Ogal, Sebastian L. Johnston, Peter Klein, Roland Schoop

**Affiliations:** 1 Pediatric Clinic, Brunnen, Switzerland; 2 National Heart and Lung Institute, Imperial College London, London, UK; 3 d.s.h. Statistical Services GmbH, Rohrbach, Germany; 4 A Vogel AG, Grünaustrasse 4, 9325 Roggwil, TG Switzerland

**Keywords:** Respiratory tract infections, Prevention, Echinacea, Complications, Antibiotics, Antiviral

## Abstract

**Background:**

In children, up to 30% of viral respiratory tract infections (RTIs) develop into bacterial complications associated with pneumonia, sinusitis or otitis media to trigger a tremendous need for antibiotics. This study investigated the efficacy of *Echinacea* for the prevention of viral RTIs, for the prevention of secondary bacterial complications and for reducing rates of antibiotic prescriptions in children.

**Methods:**

Echinaforce® Junior tablets [400 mg freshly harvested *Echinacea purpurea* alcoholic extract] or vitamin C [50 mg] as control were given three times daily for prevention to children 4–12 years. Two × 2 months of prevention were separated by a 1-week treatment break. Parents assessed respiratory symptoms in children via *e*-diaries and collected nasopharyngeal secretions for screening of respiratory pathogens (Allplex® RT-PCR).

**Results:**

Overall, 429 cold days occurred in N_ITT_ = 103 children with *Echinacea* in comparison to 602 days in N_ITT_ = 98 children with vitamin C (*p* < 0.001, Chi-square test). *Echinacea* prevented 32.5% of RTI episodes resulting in an odds ratio of OR = 0.52 [95% CI 0.30–0.91, *p* = 0.021]. Six children (5.8%) with *Echinacea* and 15 children (15.3%) with vitamin C required 6 and 24 courses of antibiotic treatment, respectively (reduction of 76.3%, *p* < 0.001). A total of 45 and 216 days of antibiotic therapy were reported in the two groups, respectively (reduction of 80.2% (*p* < 0.001). Eleven and 30 events of RTI complications (e.g., otitis media, sinusitis or pneumonia) occurred with *Echinacea* and vitamin C, respectively (*p* = 0.0030). *Echinacea* significantly prevented influenza (3 vs. 20 detections, *p* = 0.012) and enveloped virus infections (29 vs. 47 detections, *p* = 0.0038). Finally, 76 adverse events occurred with *Echinacea* and 105 events with vitamin C (*p* = 0.016), only three events were reported possibly related with *Echinacea*.

**Conclusions:**

Our results support the use of Echinacea for the prevention of RTIs and reduction of associated antibiotic usage in children.

*Trial registration* clinicaltrials.gov, NCT02971384, 23th Nov 2016.

**Supplementary Information:**

The online version contains supplementary material available at 10.1186/s40001-021-00499-6.

## Introduction

Respiratory tract infections (RTIs) continue to be the main reason for prescription of antibiotics [1, 2]. Due to a yet underdeveloped immune system, children are primarily affected by RTIs and tend to develop complications including bronchitis, pneumonia, sinusitis and especially otitis media [3]. As many as 12 RTIs may occur per child every year and a complication rate of 30% results in multiple occasions where the use of antibiotic is considered or at least debated [4–6].

A wide range of viral pathogens induce RTIs and some are reported to actively suppress immune functions (i.e., interferon production by influenza or respiratory syncytial virus (RSV) [7, 8]. In consequence, primary viral infections may be complicated by secondary bacterial infections [9, 10]. Hence, the fear of secondary bacterial infections and uncertainty pertaining to diagnosis are the main motives for dispensation of antibiotics for RTIs [11]. In individual cases in children, the scientific rationale to avoid antibiotics is often drowned out by parent’s expectations for medicinal help for their child and physicians desire to “stay on the safe side” [12]. For this reason, antibiotic stewardship programs have so far shown limited success only [13, 14]. An alternative strategy would be to impact the upstream root cause of secondary bacterial infections, the viral RTIs that initiate them. The most rational strategy would be to do this in the most affected population, i.e., in children.

Hence, the present study investigated the efficacy of Echinacea for the prevention of viral RTIs, for the prevention of secondary bacterial complications of these viral RTIs and for reducing rates of antibiotic prescriptions in children.

A child-friendly Echinacea formulation (Echinaforce® Junior tablets, EFJ) was developed by A. Vogel, Switzerland. The novel galenic form essentially aims to mask the tingling of Echinacea, often negatively perceived by children. In this study, EFJ was administered as approved by the Swiss Agency for Therapeutic Products (Swissmedic) and was compared with vitamin C (VC) as reference treatment.

## Methods

### Study design

This randomized, controlled and blinded study was carried out at 13 general practices and pediatric clinics in Switzerland during the winter season 2016/2017 (clinicaltrials.gov number NCT02971384). A total of 200 healthy children aged 4–12 years were to be enrolled upon informed consent from their parents/legal guardian. At visit 1 (V1) children were allocated to Echinacea or vitamin C group according to an electronically generated randomization list (1:1 distribution, RanCode3.6, IDV Gauting) to receive the corresponding prevention sufficient for 2 months therapy. Following a 1-week treatment break (as stipulated in the patient information), an intermediate visit (V2) was scheduled to dispense a fresh supply of the same medication for another 2 months of prevention. Previously taken concomitant medication could be continued without restriction, unless they lead to exclusion at V1 (see below). Parents were requested to abstain from giving their children further Echinacea or vitamin C products and to try to avoid other cold remedies. Upon observation of new respiratory symptoms suggesting a cold in their child, parents contacted the physician for confirmation of diagnosis and started with symptom assessment on *e*-diaries. On the same day, parents collected nasopharyngeal secretions for screening of respiratory pathogens. No rescue medication was provided. This study was approved by local and lead ethic committees (EKNZ, Switzerland, 17th October 2016) of the respective districts as well as by the national authority (Swissmedic) and strictly followed the regulations from ICH-GCP and the declaration of Helsinki (version 21^st^ October 2013). A study coordinator was available to answer questions on technical study conduct. Instructions were issued to parents concerning the detection and rating of cold symptoms in their children, derived from previously published methods by Taylor et al. [15].

### Study participants

Healthy children between 4 and 12 years of age inclusive were screened and excluded from participation if they were taking antimicrobial substances, salicylates or immunosuppressives, or if they had known diabetes mellitus, actively treated atopy or asthma, metabolic, autoimmune, degenerative or malabsorption disorders, liver or kidney disease or other severe health condition (cystic fibrosis or bronchopulmonary dysplasia), or allergy to the ingredients of the investigational medicinal products.

### Investigational medicinal products (IMPs)

Verum tablets delivered 400 mg of *Echinacea purpurea* extract (Echinaforce®, EFJ) comprising 380 and 20 mg of ethanolic extract from freshly harvested above-ground plant parts and roots, respectively (drug to extraction solvent ratio of 1:12 and 1:11, extractant 65% EtOH (V/V)). EFJ tablets were administered for prevention according to the approved package leaflet three times daily, delivering a total daily dose of 1200 mg, which corresponds to 50% of the adult prevention dose. No further adjustment of posology to the age applied and all children received the same prevention regimen. Control tablets contained 50 mg vitamin C (20 mg ascorbic acid and 36 mg calcium ascorbate) and the same amount of natural orange flavor like verum for masking. Both tablets looked, smelled and tasted similar. For the present study, vitamin C (VC) was considered as reference/control treatment of negligible activity—similar to placebo [16]. The tablets were administered 3 times daily delivering a total daily dose of 150 mg vitamin C, following recommendations from European Food Safety Authority (EFSA). At the final visit, investigators and parents were asked whether they guessed which treatment they were randomized to and if so, what medication they believed the child had received. Four vials of medication, each covering one month of prevention were identically labeled with batch numbers 046001A/045181B and according to Annex 13 of ICH-GCP and packed in identical looking patient boxes. Compliance with respect to IMP intake was assessed by weighing the content of returned medication at the final visit.

### Assessments

Presence and severity of the following symptoms ‘runny nose’, ‘blocked nose’, ‘sneezing’, ‘headache and aching limbs’, ‘sore throat’, ‘cough’, ‘chilliness’, ‘disturbed sleep quality of the child’, ‘malaise’, ‘need for additional care-giving’ were rated as “absent” [0], “mild” [1], “moderate” [2], “severe” [3] or “severity not assessable” [X] [17]. Temperature was recorded daily using an electronic thermometer (Medisana TM750, Neuss, Germany). Symptom ratings were entered contemporaneously into an internet-based electronic e-diary for up to 10 days or until resolution (absence of symptoms).

During acute infections, parents collected a sample of nasopharyngeal secretion from the child by inserting a mid-turbinate flocked nasal swab (Copan, S.p.a., Brescia, IT) which was analyzed by semi-quantitative RT-PCR for viral nucleic acid (Allplex, Roche, Switzerland) by a certified central laboratory (Labor Risch, Buchs, Switzerland). The following pathogens were screened: coronavirus (CoV) types 229E, NL63, OC43, rhinoviruses (HRV), adenoviruses (AdV), enteroviruses (HEV), respiratory syncytial virus (RSV) A and B, influenza viruses (Flu) A/H3/pdm09 and B, bocaviruses 1–4 (HBoV), metapneumovirus (MPV) and parainfluenza viruses (PIV) 1–4.

### Statistical analysis

The primary outcome was the cumulative number of days with cold symptoms during the 4-month prevention. Cold days were defined as any day on which cold symptoms were recorded anything other than “absent”. The ratio of cumulative cold days (F_VitC_/F_EFJ_) in both groups was compared with the ratio of the corresponding patient numbers in the intention-to-treat (ITT) population (N_VitC_/N_EFJ_). The χ^2^-distribution was used to test whether F_VitC_/F_EFJ_ and N_VitC_/N_EFJ_ differed significantly between the applied treatments (level of significance α = 0.05, two-sided). Presuming comparable group sizes (N_VitC_/N_EFJ_ ≈ 1) and a difference in cold days of F_VitC_/F_EFJ_ = 1.15 a sample size of N = 200 was sufficient to demonstrate superiority of EFJ over VC on basis of α < 0.05 with a power of β > 80%. Some patients entered the trial with active cold symptoms, which were however not included in the analysis.

Further variables included the incidence of RTI episodes as defined by Jackson [18], also considering respiratory infections identified as adverse events or by virus detection. RTI complications were obtained from adverse event reports, co-morbidities reported on study visits as well as indications from concomitant medications (e.g., antibiotics). RTI complications included the following medically confirmed conditions: tonsillitis, (obstructive) bronchitis, pneumonia, (rhino-) conjunctivitis, laryngotracheitis, sinusitis, otitis media, scarlet fever or (streptococcal) pharyngitis [19]. Antibiotic treatments were gathered from entries as concomitant medication (coded after WHO ATC 2016) reported during study visits, by investigators in medical records or by parents in e-diaries.

Single cold symptoms and the total symptom score (TSSc) were determined and area-under-curve calculated. Time to resolution of episodes (all symptoms rated as “absent”) was illustrated using a Kaplan–Meier survival graphic and comparison between treatments analyzed using log-rank test. Further variables included pathogen screening and the question to parents whether the therapy “barely changed” or “(relevantly) improved” the child’s status regarding resistance to infections. Tolerability and efficacy of medication were subjectively rated as “poor” [0], “moderate” [1], “good” [2] or “very good” [3] at exclusion visit. Adverse events (AEs) were closely monitored by routine calls and were judged regarding severity, relationship with IMP and duration using the method applied by Taylor et al. [15]. Children who sucked or chewed EFJ might have recognized the IMP as verum due to the popularity of the product. Here, parents were requested not to exceed the maximal approved daily dosage for treatment of acute cold symptoms. An additional sensitivity analysis excluded those unblinded participants to investigate the robustness of results. The safety population comprised all subjects having administered at least one dose of IMP, the intention-to-treat (ITT) population included all subjects reporting at least one assessment of efficacy and/or safety and finally the per-protocol (PP) population included patients from the ITT population with 80% compliance in terms of IMP intake, no premature study termination and complying with in- and exclusion criteria. Unless otherwise stated, results for the ITT population are presented for this study.

## Results

### Patient disposition

**Fig. 1:**
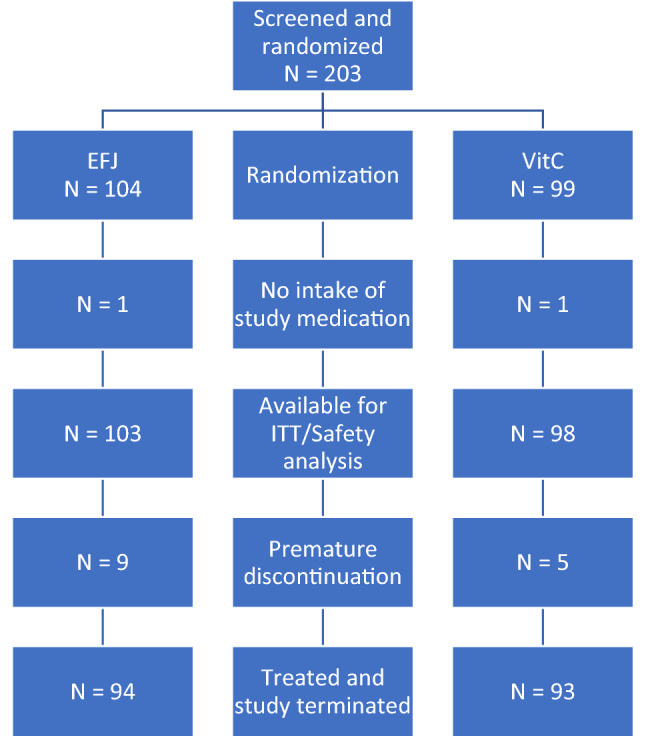
Flow diagram of disposition of patients

Overall, 203 healthy children, 4 to 12 years old were screened and recruited by 13 pediatricians and general practitioners in Switzerland from November 2016 to August 2017. No intake of study medication was noted in one participant in each group resulting in a safety and ITT population comprising N = 103 patients allocated to EFJ and N = 98 to VC. 187 patients (92.1%) completed the study. Another 14 randomized subjects (6.9%) discontinued the study prematurely, 4 (EFJ) and 3 (VC) due to administrative reasons, 2 and 1 not appearing to study visits, 2 with EFJ withdrew informed consent and one subject in each group discontinued due to an adverse event (Fig. 1).

### Baseline characteristics

Patient characteristics were similar in the treatment groups as shown in Table 1. Boys and girls were equally distributed between groups. A good proportion of children (35.7%) were below 6 years of age. No statistically or medically relevant differences were observed between groups. Only 5% of included children had been administered Echinacea or vitamin C in the 3 months prior inclusion, the remainder were therapy-naïve. A minority of 3.9% and 4.1% of children allocated to EFJ and VC had been vaccinated to influenza prior inclusion into the study (*p* = 1.00, Fisher’s exact test), whereas 48.5% and 40.8% in the same groups had received pneumococcal vaccination (*p* = 0.321, Fisher’s exact test). On average children spent 4.1 months in the study, exactly as scheduled.

**Table 1:** Patient characteristics presenting at baseline

	Safety/ITT population		Per protocol population	
	EFJ (N = 103)	VC (N = 98)	EFJ (N = 64)	VC (N = 75)
Age classes **( **years**)**				
4–6 years	41 (39.8%)	32 (32.6%)	24 (37.5%)	28 (37.3%)
> 7 years	62 (60.2%)	66 (67.4%)	40 (62.5%)	47 (62.7%)
Mean (SD)	7.8 (2.74)	8.1 (2.71)	8.1 (2.81)	7.9 (2.69)
Height [cm] Mean (SD)	127.0 (16.35)	130.1 (16.37)	128.7 (16.42)	128.2 (16.33)
Body weight [kg] Mean (SD)	28.1 (10.60)	29.1 (11.49)	28.8 (10.47)	28.1 (11.21)
Previous annual RTIs (SE)	3.2 (0.23)	3.3 (0.27)	3.2 (0.30)	3.4 (0.34)
Previous annual complicated RTIs (SE)	0.6 (0.09)	0.6 (0.09)	0.7 (0.13)	0.5 (0.08)
Attendance of school or kindergarten [yes (SD)]	93 (90.3%)	92 (93.9%)	60 (93.8%)	70 (93.3%)

The age in years was calculated according to the formula: year of visit 1 – year of birth

*SD* standard deviation, *SE* standard error mean, *EFJ* Echinaforce Junior tablets, *VC* vitamin C tablets

### Evaluation of efficacy

#### Cumulative cold days

The cumulative number of cold days was 429 days for EFJ (N = 103 subjects) and 602 days for VC (N = 98 subjects). The ratio of the number of subjects N_VC_/N_EFJ_ = 0.95 to the number of cumulative cold days F_VC_/F_EFJ_ = 1.40 indicated 47% more symptom days for VC and a statistically significant superiority for EFJ (ITT, *p* < 0.0001, Chi-square test). Results in the per-protocol population were with a ratio of 1.81 more symptom days even more pronounced (PP, *p* < 0.0001, Chi-square test).

#### RTI incidence

With EFJ and VC 38.8% and 55.1% of children experienced 61 and 86 cold episodes in contrast to 61.2% and 44.9%, who stayed free of colds, respectively. *Echinacea* prevented 32.5% of RTI episodes resulting in an odds ratio OR = 0.52 [95% CI 0.30–0.91, *p* = 0.021]. In the PP population an OR = 0.36 [95% CI 0.18–0.71, *p* = 0.003] was reached. Per subject 0.6 ± 0.89 (SD) cold episodes were registered on EFJ prevention and 0.9 ± 1.03 episodes with VC (ITT, *p* = 0.023, Mantel–Haenszel test). A low number needed to treat (NNT) of 4 was calculated to prevent one RTI with EFJ.

#### Determination of pathogens

Nasopharyngeal samples were obtained from subjects with EFJ (N_ITT_ = 103) yielding 57 positive virus detections (55.3%) in contrast to 72 positive detections (67.3%) in the VC group (N_ITT_ = 98, *p* = 0.0074). RT-PCR analysis results for the ITT and PP collective are given as supplementary material (Additional file 1: Appendix S1). No significant difference was observed for AdV or HRV infections (*p* > 0.1). However, with EFJ and VC a total of 29 and 47 samples, respectively, were positively tested for enveloped viruses including RSV, CoV, MPV, PIV, HBoV and influenza (*p* = 0.0038; Chi-square test). The mean number of enveloped viruses detected per patient was 0.3 for the EFJ group and 0.5 for the VC group (*p* = 0.118). On the level of single pathogens, EFJ significantly prevented influenza from 20 detections with VC to 3 detections in the EFJ group (*p* = 0.012).

#### RTI complications and antibiotic prescriptions

Next, we investigated whether prevention of RTI episodes with EFJ would result in fewer RTI complications. In fact, there were 52.5% fewer patients (N = 10, 9.7%) with RTI complications in the EFJ group, in comparison to 20 patients (20.4%) with VC (*p* = 0.047). The cumulative number of RTI complications was 11 (10.7%) for the EFJ group and 30 (30.6%) for VC, indicating a relative and absolute risk reduction of 65.0% and 19.9% (*p* < 0.0030, Chi-square test).

Six (5.8%) children in the EFJ group each required a single antibiotic treatment for 45 days overall, in comparison to 15 (15.3%) children with 24 prescriptions (24.5%) on 216 days in the vitamin C group. Results indicate a relative (RRR) and absolute risk reduction (ARR) for antibiotic prescriptions of 76.3% and 18.7%, respectively (*p* = 0.0012). Days with antibiotics were reduced by as much as 80.2% (*p* < 0.0001). Five children needed to be treated with EFJ to prevent each of one RTI complication and one course of antibiotic treatment (both NNT = 5). Compared with VC, prevention with EFJ reduced antibiotic treatment days by as much as 171 days in a sample of 103 children, indicating a 1.67-day reduction in antibiotic treatment per child taking EFJ prevention for 4 months, equivalent to 4.98 days per child and year (*p* < 0.0001).

#### Severity and duration of episodes

Time until symptom resolution was illustrated using an analysis according to Kaplan–Meier. A mean duration of RTI episodes of 5.7 ± 2.74 days and 7.1 ± 3.38 days was calculated indicating a reduction in duration of 1.4 days with EFJ (ITT, *p* = 0.018, Wilcoxon test). At day 10, 29 VC-treated episodes remained symptomatic in comparison to 15 episodes with EFJ. Until cessation of symptoms the aggregated symptom scores of individual RTI episodes (area-under-curve, AUC) amounted to 35.5 ± 28.76 and 53.5 ± 42.80 indicating a 33.6% reduction with EFJ (*p* = 0.011, Wilcoxon test). Days with fever were strongly reduced with EFJ from a mean of 4.9 ± 6.61 days to 1.6 ± 4.34 days (*p* < 0.001) as were other symptoms typical of flu-like illnesses: coughing, headache or aching limbs (*p* < 0.05, Wilcoxon test, data not shown).

#### Subjective judgement of efficacy, acceptance, blinding and compliance

After 4 months prevention significantly more parents (89.8%) stated that EFJ “*improved*” or “*significantly improved*” the resistance status of their child in comparison to VC with 70.8% (*p* = 0.010). Acceptance was consequently rated high and 91.8% acknowledged that they would take EFJ again, whereas this was the case in only 76% of VC recipients (*p* = 0.030). Finally, blinding was efficient and the same high proportion of physicians and parents could not ascertain the allocated treatment in both EFJ and VC groups (86.7% vs 86.5 and 85.7 vs 88.5%, *p* > 0.6). A sensitivity analysis excluded children whose physicians and/or parents correctly guessed their allocated treatment and showed that above results remained robust, positive and significant. Participants were highly compliant and during 4 months consumed the same amount of tablets in both groups, corresponding to 88.2% (27.30) and 93.4% (25.96) of the theoretically calculated amount of EFJ and VC for 4 months, i.e., 108 g of tablets (ITT population, *p* > 0.1).

#### Tolerability and safety

Overall, 98.0% of parents and investigators judged the tolerability of EFJ as “*(very-) good*”, similar to the control group with 95.9% and 96.8%, respectively. No significant difference between EFJ and control was identified (*p* > 0.5).

As seen in Table 2, there were no noticeable differences in the number of patients with adverse events, in the number of patients with drug-related AEs (ADRs), AEs leading to study termination and occurrence of SAEs (*p* > 0.2). Overall, adverse events occurred significantly less frequent with EFJ, which was mainly due to the reduced number of RTI complications mentioned above (*p* = 0.0157). Two and three patients (2.0% [VC] and 2.9% [EFJ]) reported an adverse drug reaction with possible causal relationship to the study medication. These were a case each of stomatitis, vomiting, diarrhea, urticaria and choking, respectively. Allergic and hypersensitivity reactions including rash or urticaria occurred with the same frequency of 5.8 and 6.1% in both groups. One serious adverse event in form of a humeral fracture and a distal tibia fracture was reported in each group, but these were not reported related with intake of study drug.

**Table 2:** Safety variables

	EFJ (N = 103) [%]	VC (N = 98) [%]	P value
Adverse events (AEs)	76 [73.8%]	105 [107.1%]	0.016
Patients with AEs	51 [49.5%]	45 [45.9%]	0.672
Adverse drug reactions	3 [2.9%]^a^	2 [2.0%]^b^	1.000
AEs causing study termination	0 [0%]	2 [2.0%]^c^	0.236
Serious adverse events (SAE)	1 [1.0%]^d^	1 [1.0%]^e^	1.000

^a^Diarrhea, mild urticaria, choking

^b^Stomatitis, vomiting

^c^Otitis media, stomatitis

^d^Humeral fracture

^e^Distal tibia fracture

## Discussion

Antibiotic stewardship promotes the judicious use of antibiotics and action plans aim to reduce their overuse [20]. However, a 6-year national campaign in the United Kingdom produced only a modest change in their use for the most frequent indication RTIs. As a consequence, 67% of general practitioners continued prescribing antibiotics for RTIs, with the highest prevalence (90%) for chest infections, 80% for ear infections and 60% for a sore throat [13]. A 22% decline was achieved by attempts in Sweden but over 80% of otitis media and 70% of sinusitis episodes continued to be treated with antibiotics. [14] Once infections are established, uncertainties in diagnosis and patient’s expectation in the individual case undermine any sophisticated awareness programs for the sake of the community. This conflict is further accentuated in the pediatric setting when dealing with a highly vulnerable population [21]. In our study, we followed an alternative approach to try to prevent rather than treat RTIs as the root cause of RTI complications and antibiotic use, and employed a child-friendly formulation containing Echinacea.

We report that use of Echinacea, compared to our control of vitamin C resulted in significant prevention of cold days and respiratory tract infections by up to 32.5%. Benefits included a specific prevention of enveloped virus infections, including RSV and in particular a marked reduction of influenza virus infections. RTI complications were reduced by 65% and lastly, antibiotic prescriptions were reduced by up to 76.3% or by 171 treatment days, which for 103 children taking Echinacea for 4 months, equates to a reduction in days of antibiotic therapy of 4.98 days per child per year. Low numbers needed to treat (NNTs ≤ 5) were found throughout, which is not only a result of treatment benefits, but also of the frequency of the studied medical entity (i.e., RTIs and their complications).

Our figures are in agreement with data from the literature. A RTI complication risk of 30% has repeatedly been observed in children. In contrast to this, the numbers for EFJ were very low at 11%. [3] Very similar effects were finally seen on the numbers of RTI complications and antibiotic prescriptions, indicating a clear correlation between the two entities. The reduction of antibiotics is thus unlikely derived from any non-specific prevention (i.e., topical or gastro-intestinal complaints) by chance, but due to specific prevention of respiratory illnesses. The overall prescription rate of 5.8% in the EFJ group compares with data from primary care physicians in Switzerland applying antibiotics to 20.4% of respiratory tract infections and between 41.5 and 69.6% for RTI complications like acute bronchitis or otitis media. In our control group, as much as 24.5% received prescriptions, which correlates well with increased RTI complications in this group and otherwise reported figures on antibiotic use. [22, 23].

Overall results on preventive benefits of Echinacea are highly heterogenous [19, 24], which has been explained by quality variation between investigated products. The differentiation of lipophilic and hydrophilic preparations seems to unravel this complexity to a certain degree. [25] A meta-analysis of randomized controlled trials reported that lipophilic extracts from (freshly harvested) *Echinacea purpurea* reduced recurrent respiratory tract infections by 35% and complications thereof by up to 50% when administered for a period of 2–4 months in adults. [19] This in sharp contrast to hydrophilic preparations like pressed-juices, which showed no significant benefit. Hence, the extractant polarity appears important in obtaining pharmacologically active substances from the medicinal plant. For instance, alkylamide derivatives are optimally extracted under lipophilic conditions and from freshly harvested, rather than dried plant material. [26] Alkylamides are known to interact with the human endocannabinoid system and exhibit anti-inflammatory effects. [27] Alcoholic extracts from *Echinacea purpurea* were further shown to inhibit infectivity of a variety of enveloped respiratory viruses including influenza, RSV or coronaviruses via blocking of viral docking receptors but this activity could so far not be attributed to a particular marker substance in the extract. [28, 29].

Reduction of antibiotics by Echinacea has previously been observed in cold prevention studies but has never been the subject of specific research. A 50% reduction of prescriptions and 3.4 ± 5.6 and 6.5 ± 8.0 mean days of antibiotic use with Echinacea in comparison with placebo were documented by Cohen during 3 months prevention in a group of children 1 to 5 years of age. [30] Another 3-month open, observational study administered Echinacea to adults with acute RTIs and found 4.4% antibiotic prescriptions in comparison to 14.3% with standard treatment. [31] Both studies were included in the above-mentioned meta-analysis, which found a clear reduced risk of recurrent RTIs and secondary RTI complications with Echinacea use, but only a single study [30] reporting a reduction of antibiotics. [19] Our results are in good agreement with these previous reports and provide further evidence of a substantial reduction in antibiotic usage with Echinacea from a randomized, controlled and blinded study in a pediatric setting.

As already reported by Jawad and colleagues, Echinacea exhibits a high specificity towards enveloped respiratory viruses in vitro and in vivo, which however strongly depends on the manufacturing process. [32–35] Results were confirmed in the present study, and a strong reduction was evident for RSV or influenza virus infections, some of which represent frequent triggers for complications and admissions in very young children (< 5 years). [12, 36, 37] Our results together with data from Cohen may thus have implications relating to Echinacea use in pre-school children, although our exclusion of children younger than 4 years old does not permit us to make a direct recommendation for this population.

Originally, our study was not designed as RCT to determine the extent of antibiotic reduction as primary parameter but of days with cold instead. Sample size estimation was calculated accordingly. Nevertheless, the study was conceptualized and large enough to determine an effect on complications and on antibiotics similar to that observed in adults with a power of > 90%. Twelve to 15% of participants correctly guessed the treatment group they were allocated to and might have introduced a potential bias. Exclusion of those unblinded participants, however did not change the significance of the outcomes, which further corroborates the robustness of findings. This might be explained by the fact that none of the participants received pure placebo and even the group with vitamin C may have expected some benefit. Vitamin C was chosen as reference treatment right for this purpose and to motivate parents for participation in the study. Scientifically, the value of vitamin C for cold prevention has not been substantiated convincingly. A meta-analysis by Hemilae showed that only higher daily doses of > 1000 mg enhanced the reduction of cold duration to 18.1% (9% to 27%) from 14.2% (7 to 22%), produced by dosages > 200 mg in children below 18 years of age. [16] Dosages below 200 mg, as used in the present study, were not even considered in the analysis. Moreover, data on prevention or treatment of RTI complications like pneumonia are inconclusive. [38] In our study, we therefore considered vitamin C as reference of negligible efficacy (i.e., placebo); whereas, even a moderate positive effect of the control treatment would only have further increased benefits for Echinacea. We therefore think we have applied a highly conservative approach. It would be very interesting to study preventive benefits of Echinacea in vaccinated versus unvaccinated children in future clinical observations, whereas this study was ultimately too small to further investigate effects in a subgroup of included children.

The safety of Echinacea was very good with very low numbers of adverse events thought possibly related to study medication, and no differences between EFJ and VC control in this respect was found. The overall number of adverse events was significantly (*p* = 0.016) lower with EFJ than with vitamin C, consistent with the reduction of RTIs, complications of RTIs and antibiotic usage. An increased risk for rashes (7.1 vs 2.7%) was reported [15] for an Echinacea pressed-juice preparation, however this was not observed for the hydroethanolic extract used in the current study with 1.9 and 2.0% for EFJ and VC, respectively.

Altogether, our results strongly suggest the use of Echinacea (i.e., Echinaforce Junior tablets) for the long-term prevention of RTIs, cold days, influenza and other enveloped virus infections, RTI complication and antibiotic usage in children 4 – 12 years. Its effect in preventing enveloped virus infections (including RSV) as well as a prior study showing efficacy of Echinacea in children 1–5 years of age [30] suggests the use in younger children as well.

## Supplementary Information

**Additional file 1.** Results from RT-PCR Virus Analysis for Intention-to-treat and Per Protocol Collective.

## Data Availability

Data supporting the conclusions of this article are included in this article.
