# Supplementary material for: Echinacea reduces antibiotic usage in children through respiratory tract infection prevention: a randomized, blinded, controlled clinical trial
Source: Eur J Med Res. 2021 Apr 8;26:33. doi: 10.1186/s40001-021-00499-6 (PMC8028575; doi:10.1186/s40001-021-00499-6)
Supplement: Supplementary file 1 — Additional file 1. Results from RT-PCR Virus Analysis for Intention-to-treat and Per Protocol Collective. [file 40001_2021_499_MOESM1_ESM.docx]

**Appendix 1:** Results from RT-PCR Virus Analysis for Intention-to-treat and Per Protocol Collective

| Pathogen | **EFJ** (N_ITT_=103) | **VC** (N_ITT_=98) | p-value | **EFJ** (N_PP_=64) | **VC** (N_PP_=75) | p-value |
| --- | --- | --- | --- | --- | --- | --- |
| Adenovirus [AdV] | 3 | 3 |  | 1 | 2 |  |
| Coronavirus [229E/OC43/NL63] | 13 | 11 |  | 8 | 10 |  |
| Influenza [A/H3/pdm09 and B] | 3 | 20 |  | 3 | 16 |  |
| Parainfluenza [PIV1-4] | 1 | 3 |  | 0 | 3 |  |
| Respiratory Syncytial Virus [RSVA/B] | 10 | 11 |  | 7 | 9 |  |
| Metapneumovirus [MPV] | 1 | 1 |  | 1 | 1 |  |
| Bocavirus [HBoV] | 1 | 1 |  | 0 | 1 |  |
| Rhinovirus/Enterovirus [HRV/HEV] | 25 | 22 |  | 15 | 21 |  |
|  |  |  |  |  |  |  |
| Membranous | 29 | 47 | P=0.0038 | 19 | 40 | P=0.0049 |
| Non-membranous | 28 | 25 | P=0.7877 | 16 | 23 | P = 0.4586 |
| Sum | 57 | 72 | P=0.0074 | 35 | 63 | P=0.0002 |

**Incidences - ITT**

| \| \| **Membranous ITT** \| \| --- \| \| \| --- \| --- \| | | \| **Table of event by group** \| \| \| \| \| --- \| --- \| --- \| --- \| \| **event** \| **group** \| \| \| \| **1** \| **2** \| **Total** \| \| **0** \| \| 74 \| \| --- \| \| 71.84 \| \| \| 51 \| \| --- \| \| 52.04 \| \| \| 125 \| \| --- \| \| \| **1** \| \| 29 \| \| --- \| \| 28.16 \| \| \| 47 \| \| --- \| \| 47.96 \| \| \| 76 \| \| --- \| \| |
| --- | --- | --- | --- | --- | --- | --- | --- | --- | --- | --- | --- | --- | --- | --- | --- | --- | --- | --- | --- | --- | --- | --- | --- | --- | --- | --- | --- | --- | --- | --- | --- | --- | --- |
|  |  |  |

| **Statistic** | **DF** | **Value** | **Prob** |
| --- | --- | --- | --- |
| **Chi-Square** | 1 | 8.3760 | 0.0038 |

| \| \| **Non-Membranous**  **ITT** \| \| --- \| \| \| --- \| --- \| | | \| **Table of event by group** \| \| \| \| \| --- \| --- \| --- \| --- \| \| **event** \| **group** \| \| \| \| **1** \| **2** \| **Total** \| \| **0** \| \| 75 \| \| --- \| \| 72.82 \| \| \| 73 \| \| --- \| \| 74.49 \| \| \| 148 \| \| --- \| \| \| **1** \| \| 28 \| \| --- \| \| 27.18 \| \| \| 25 \| \| --- \| \| 25.51 \| \| \| 53 \| \| --- \| \| |
| --- | --- | --- | --- | --- | --- | --- | --- | --- | --- | --- | --- | --- | --- | --- | --- | --- | --- | --- | --- | --- | --- | --- | --- | --- | --- | --- | --- | --- | --- | --- | --- | --- | --- |
|  |  |  |

| **Statistic** | **DF** | **Value** | **Prob** |
| --- | --- | --- | --- |
| **Chi-Square** | 1 | 0.0725 | 0.7877 |

| \| \| **Frequency** \| \| --- \| \| \| --- \| --- \| | | \| **Table of event by group** \| \| \| \| \| --- \| --- \| --- \| --- \| \| **event** \| **group** \| \| \| \| **1** \| **2** \| **Total** \| \| **0** \| \| 46 \| \| --- \| \| 44.66 \| \| \| 26 \| \| --- \| \| 26.53 \| \| \| 72 \| \| --- \| \| \| **1** \| \| 57 \| \| --- \| \| 55.34 \| \| \| 72 \| \| --- \| \| 73.47 \| \| \| 129 \| \| --- \| \| |
| --- | --- | --- | --- | --- | --- | --- | --- | --- | --- | --- | --- | --- | --- | --- | --- | --- | --- | --- | --- | --- | --- | --- | --- | --- | --- | --- | --- | --- | --- | --- | --- | --- | --- |
|  |  |  |

| **Statistic** | **DF** | **Value** | **Prob** |
| --- | --- | --- | --- |
| **Chi-Square** | 1 | 7.1798 | 0.0074 |

**Incidences - PPROT**

| \| \| **Membranous PPROT** \| \| --- \| \|  \| \| \| --- \| --- \| --- \| | | \| **Table of event by group** \| \| \| \| \| --- \| --- \| --- \| --- \| \| **event** \| **group** \| \| \| \| **1** \| **2** \| **Total** \| \| **0** \| \| 45 \| \| --- \| \| 70.31 \| \| \| 35 \| \| --- \| \| 46.67 \| \| \| 80 \| \| --- \| \| \| **1** \| \| 19 \| \| --- \| \| 29.69 \| \| \| 40 \| \| --- \| \| 53.33 \| \| \| 59 \| \| --- \| \| |
| --- | --- | --- | --- | --- | --- | --- | --- | --- | --- | --- | --- | --- | --- | --- | --- | --- | --- | --- | --- | --- | --- | --- | --- | --- | --- | --- | --- | --- | --- | --- | --- | --- | --- | --- |
|  |  |  |

| **Statistic** | **DF** | **Value** | **Prob** |
| --- | --- | --- | --- |
| **Chi-Square** | 1 | 7.9036 | 0.0049 |

| \| \| **Non-Membranous**  **PPROT** \| \| --- \| \| \| --- \| --- \| | | \| **Table of event by group** \| \| \| \| \| --- \| --- \| --- \| --- \| \| **event** \| **group** \| \| \| \| **1** \| **2** \| **Total** \| \| **0** \| \| 48 \| \| --- \| \| 75.00 \| \| \| 52 \| \| --- \| \| 69.33 \| \| \| 100 \| \| --- \| \| \| **1** \| \| 16 \| \| --- \| \| 25.00 \| \| \| 23 \| \| --- \| \| 30.67 \| \| \| 39 \| \| --- \| \| |
| --- | --- | --- | --- | --- | --- | --- | --- | --- | --- | --- | --- | --- | --- | --- | --- | --- | --- | --- | --- | --- | --- | --- | --- | --- | --- | --- | --- | --- | --- | --- | --- | --- | --- |
|  |  |  |

| **Statistic** | **DF** | **Value** | **Prob** |
| --- | --- | --- | --- |
| **Chi-Square** | 1 | 0.5493 | 0.4586 |

| \| \| **Sum PPROT** \| \| --- \| \| \| --- \| --- \| | \| **Table of event by group** \| \| \| \| \| --- \| --- \| --- \| --- \| \| **event** \| **group** \| \| \| \| **1** \| **2** \| **Total** \| \| **0** \| \| 29 \| \| --- \| \| 45.31 \| \| \| 12 \| \| --- \| \| 16.00 \| \| \| 41 \| \| --- \| \| \| **1** \| \| 35 \| \| --- \| \| 54.69 \| \| \| 63 \| \| --- \| \| 84.00 \| \| \| 98 \| \| --- \| \| |
| --- | --- | --- | --- | --- | --- | --- | --- | --- | --- | --- | --- | --- | --- | --- | --- | --- | --- | --- | --- | --- | --- | --- | --- | --- | --- | --- | --- | --- | --- | --- | --- | --- |

|  |
| --- |

| **Statistic** | **DF** | **Value** | **Prob** |
| --- | --- | --- | --- |
| **Chi-Square** | 1 | 14.2676 | 0.0002 |

**Incidences CT - ITT**

| \| \| **Membranous ITT** \| \| --- \| \| \| --- \| --- \| | | \| **Table of event by group** \| \| \| \| \| --- \| --- \| --- \| --- \| \| **event** \| **group** \| \| \| \| **1** \| **2** \| **Total** \| \| **0** \| \| 73 \| \| --- \| \| 70.87 \| \| \| 49 \| \| --- \| \| 50.00 \| \| \| 122 \| \| --- \| \| \| **1** \| \| 30 \| \| --- \| \| 29.13 \| \| \| 49 \| \| --- \| \| 50.00 \| \| \| 79 \| \| --- \| \| |
| --- | --- | --- | --- | --- | --- | --- | --- | --- | --- | --- | --- | --- | --- | --- | --- | --- | --- | --- | --- | --- | --- | --- | --- | --- | --- | --- | --- | --- | --- | --- | --- | --- | --- |
|  |  |  |

| **Statistic** | **DF** | **Value** | **Prob** |
| --- | --- | --- | --- |
| **Chi-Square** | 1 | 9.1722 | 0.0025 |

| \| \| **Non-Membranous**  **PPROT** \|  \|  \| \| --- \| --- \| --- \| \| \| --- \| --- \| --- \| --- \| | | \| **Table of event by group** \| \| \| \| \| --- \| --- \| --- \| --- \| \| **event** \| **group** \| \| \| \| **1** \| **2** \| **Total** \| \| **0** \| \| 67 \| \| --- \| \| 65.05 \| \| \| 69 \| \| --- \| \| 70.41 \| \| \| 136 \| \| --- \| \| \| **1** \| \| 36 \| \| --- \| \| 34.95 \| \| \| 29 \| \| --- \| \| 29.59 \| \| \| 65 \| \| --- \| \| |
| --- | --- | --- | --- | --- | --- | --- | --- | --- | --- | --- | --- | --- | --- | --- | --- | --- | --- | --- | --- | --- | --- | --- | --- | --- | --- | --- | --- | --- | --- | --- | --- | --- | --- | --- | --- |
|  |  |  |

| **Statistic** | **DF** | **Value** | **Prob** |
| --- | --- | --- | --- |
| **Chi-Square** | 1 | 0.6593 | 0.4168 |

| \| \| **Sum ITT** \| \| --- \| \| \| --- \| --- \| | | \| **Table of event by group** \| \| \| \| \| --- \| --- \| --- \| --- \| \| **event** \| **group** \| \| \| \| **1** \| **2** \| **Total** \| \| **0** \| \| 37 \| \| --- \| \| 35.92 \| \| \| 20 \| \| --- \| \| 20.41 \| \| \| 57 \| \| --- \| \| \| **1** \| \| 66 \| \| --- \| \| 64.08 \| \| \| 78 \| \| --- \| \| 79.59 \| \| \| 144 \| \| --- \| \| |
| --- | --- | --- | --- | --- | --- | --- | --- | --- | --- | --- | --- | --- | --- | --- | --- | --- | --- | --- | --- | --- | --- | --- | --- | --- | --- | --- | --- | --- | --- | --- | --- | --- | --- |
|  |  |  |

| **Statistic** | **DF** | **Value** | **Prob** |
| --- | --- | --- | --- |
| **Chi-Square** | 1 | 5.9495 | 0.0147 |

**Incidences CT - PPROT**

| \| \| **Membranous PPROT** \| \| --- \| \|  \| \|  \| \|  \| \| \| --- \| --- \| --- \| --- \| --- \| | | \| **Table of event by group** \| \| \| \| \| --- \| --- \| --- \| --- \| \| **event** \| **group** \| \| \| \| **1** \| **2** \| **Total** \| \| **0** \| \| 45 \| \| --- \| \| 70.31 \| \| \| 35 \| \| --- \| \| 46.67 \| \| \| 80 \| \| --- \| \| \| **1** \| \| 19 \| \| --- \| \| 29.69 \| \| \| 40 \| \| --- \| \| 53.33 \| \| \| 59 \| \| --- \| \| |
| --- | --- | --- | --- | --- | --- | --- | --- | --- | --- | --- | --- | --- | --- | --- | --- | --- | --- | --- | --- | --- | --- | --- | --- | --- | --- | --- | --- | --- | --- | --- | --- | --- | --- | --- | --- | --- |
|  |  |  |

| **Statistic** | **DF** | **Value** | **Prob** |
| --- | --- | --- | --- |
| **Chi-Square** | 1 | 7.9036 | 0.0049 |

| \| \|  \| \| --- \| \|  \| \|  \| \| **Non-Membranous PPROT** \| \| \| --- \| --- \| --- \| --- \| --- \| | | \| **Table of event by group** \| \| \| \| \| --- \| --- \| --- \| --- \| \| **event** \| **group** \| \| \| \| **1** \| **2** \| **Total** \| \| **0** \| \| 42 \| \| --- \| \| 65.63 \| \| \| 48 \| \| --- \| \| 64.00 \| \| \| 90 \| \| --- \| \| \| **1** \| \| 22 \| \| --- \| \| 34.38 \| \| \| 27 \| \| --- \| \| 36.00 \| \| \| 49 \| \| --- \| \| |
| --- | --- | --- | --- | --- | --- | --- | --- | --- | --- | --- | --- | --- | --- | --- | --- | --- | --- | --- | --- | --- | --- | --- | --- | --- | --- | --- | --- | --- | --- | --- | --- | --- | --- | --- | --- | --- |
|  |  |  |

| **Statistic** | **DF** | **Value** | **Prob** |
| --- | --- | --- | --- |
| **Chi-Square** | 1 | 0.0400 | 0.8416 |

| \| **Sum PPROT** \| \| --- \| | | \| **Table of event by group** \| \| \| \| \| --- \| --- \| --- \| --- \| \| **event** \| **group** \| \| \| \| **1** \| **2** \| **Total** \| \| **0** \| \| 33 \| \| --- \| \| 35.94 \| \| \| 8 \| \| --- \| \| 10.67 \| \| \| 31 \| \| --- \| \| \| **1** \| \| 31 \| \| --- \| \| 64.06 \| \| \| 67 \| \| --- \| \| 89.33 \| \| \| 108 \| \| --- \| \| |
| --- | --- | --- | --- | --- | --- | --- | --- | --- | --- | --- | --- | --- | --- | --- | --- | --- | --- | --- | --- | --- | --- | --- | --- | --- | --- | --- | --- | --- | --- | --- | --- | --- |
|  |  |  |

| **Statistic** | **DF** | **Value** | **Prob** |
| --- | --- | --- | --- |
| **Chi-Square** | 1 | 12.7265 | 0.0004 |
